# Interdisciplinary Community-Based Support for Caregivers of Individuals Living with Dementia

**DOI:** 10.1093/geroni/igab046.2967

**Published:** 2021-12-17

**Authors:** Gina Tucker-Roghi, Jamie Escoubas, Renee Tolliver, Sarah Tucker

**Affiliations:** 1 Dominican University of California, SAN RAFAEL, California, United States; 2 Sonoma County Council on Aging, Santa Rosa, California, United States

## Abstract

Evidence indicates family caregivers of individuals living with dementia (ILwD) are at risk for diminished physical and mental health; which may decrease their quality of life and directly impact their ability to provide care. An interdisciplinary approach to self-care and skill-building for caregivers is provided in a virtual support group offered by Council on Aging in Sonoma County, CA. As part of the nonprofit’s Adult Day Program, the group is offered to client caregivers and has two goals: First, creating a community-based, long-term support system for ILwD who are aging-in-place; second, fostering a safe and supportive community for family caregivers, by providing opportunities to collaborate with peers and an interdisciplinary team that includes a Marriage and Family Therapist (MFT), an Occupational Therapist (OT), and a Recreation Therapist (the day program manager). The closed group model established through eight weekly sessions builds trusting relationships in a frame that combines: the OT client-centered and collaborative approach to problem-solving everyday challenges of caregiving, the MFT skills of creating a safe space for discussion and deeper exploration, and program staff insights regarding the ILwD’s current interests and abilities exhibited during Day Program activities. Sessions include an emotional check-in by group members; a brief overview of best-practices and common caregiving concerns related to a weekly topic; and an opportunity for caregivers to explore the integration of best-practices into daily routines, while also attending to their well-being as caregivers. Program evaluation and results related to the program’s effectiveness and implications for scalability will be discussed.

